# Relationships Between Social Support, Loneliness, and Internet Addiction in Chinese Postsecondary Students: A Longitudinal Cross-Lagged Analysis

**DOI:** 10.3389/fpsyg.2018.01707

**Published:** 2018-09-11

**Authors:** Shujie Zhang, Yu Tian, Yi Sui, Denghao Zhang, Jieru Shi, Peng Wang, Weixuan Meng, Yingdong Si

**Affiliations:** Department of Psychology, Shandong Normal University, Jinan, China

**Keywords:** social support, loneliness, Internet addiction, longitudinal cross-lagged analysis, interventions

## Abstract

Using the Internet has become one of the most popular leisure activities among postsecondary students in China. Concern about the large number of students using the Internet has led to an increase in research on the influencing factors of Internet addiction and the negative consequences caused by it. This short-term longitudinal study examined the associations among three dimensions of social support [objective support (OS), subjective support (SS), and support utilization (SU)], loneliness, and the four dimensions of Internet addiction (compulsive Internet use [CIU] & withdrawal from Internet addiction [WIA], tolerance of Internet addiction [TIA], time-management problems [TMPs], and interpersonal and health problems [IHPs]) in a Chinese sample. A total of 169 postsecondary first-year students (88 girls and 81 boys; mean age = 18.31 years) participated in the study. The questionnaire measurements were taken at the beginning of the school year (T1), 6 months later (T2), and 1 year later (T3). Cross-lagged and structural equation modeling analyses indicated that (a) OS (T1) and SU (T1) negatively predicted loneliness (T2); and loneliness (T2) negatively predicted OS (T3) and SU(T3); (b) CIU & WIA (T1) and TMPs (T1) positively predicted loneliness (T2); and loneliness (T2) positively predicted CIU & WIA (T3), TIA (T3), TMP (T3), and IHP (T3); (c) SS (T1) directly affected TIA (T3) and TMP (T3); and (d) loneliness (T2) played a mediating role in the relationships between OS (T1) and CIU (T3), OS (T1) and TMP (T3), OS (T1) and IHP (T3), and SU (T1) and IHP (T3). Finally, interventions for Internet addiction and implications for future studies were discussed.

## Introduction

According to the 40th China Internet Network Information Center [CNNIC] (2017), 751 million Chinese citizens use the Internet. Among them, 185 million are students (24.8%). Proper Internet use can contribute to the development of adolescents by promoting socialization and their ego identities (Zhang et al., 2008; Jackson et al., 2011). However, excessive Internet use is a typical negative side effect of Internet use and cannot be ignored (Ye and Zheng, 2016). No clear and unified agreement exists in the literature on the terms used for the problems associated with Internet use (Musetti et al., 2016; Musetti and Corsano, 2018). Current terms in use are Internet addiction (Block, 2008), Internet dependence (Scherer, 1997), compulsive use (Meerkerk et al., 2006), problematic Internet use (Shapira et al., 2000), pathological Internet use (Davis, 2001), and unregulated Internet usage (LaRose et al., 2003). To facilitate interpretation, “Internet addiction” is used in this paper, which refers to the “compulsive overuse of the Internet and irritable or moody behavior when deprived of it” (Douglas et al., 2008). Researchers have developed relevant scales to measure Internet addiction through the internal psychological experiences and external behavioral problems of Internet users (Suler, 1999; Chou and Hsiao, 2000; Morahan-Martin and Schumacher, 2000; Cui and Xin, 2004).

The latest research has shown that Internet addiction is a widespread problem among postsecondary students, affecting approximately 8% (2.156 million) of high school students in China (China Internet Network Information Center [CNNIC], 2017; Liu et al., 2017). Numerous studies have demonstrated that postsecondary students with Internet addiction typically experience physical health problems (Mentzoni et al., 2011; Inamori et al., 2017), poor school performance (Pang et al., 2010; Kim et al., 2017), aggressive tendencies (Staude-Müller, 2011), and problems with family relationships and social connections (Yen et al., 2007; Honnekeri et al., 2017; Longobardi et al., 2018). Therefore, investigating how postsecondary students acquire Internet addiction and developing effective intervention techniques for Internet addiction are imperative. Studies have indicated that Internet addiction is a complex psychological phenomenon influenced by numerous factors, which can be divided into the following categories: external environments (Wu, 2004; Chen et al., 2007; Wu et al., 2016), family environments (Li et al., 2014), and peer relationships (Zhou et al., 2017); psychological factors such as loneliness (Özdemir et al., 2014; Sharifpoor et al., 2017), depression (Fayazi and Hasani, 2017), negative affect and dissociation (Musetti et al., 2018) and self-esteem (Bozoglan et al., 2013; Farah et al., 2016); and physiological factors such as hormones and arousal levels (Wang, 2006). Thus, investigating the relationships between some of these variables and Internet addiction is necessary.

Because college student have ample leisure time and convenient and consistent Internet access through a range of wireless tools, modern postsecondary students often spend considerable time on the Internet, and therefore, they are more likely to experience Internet addiction (Morahan-Martin and Schumacher, 2000; Marengo et al., 2018; Settanni et al., 2018). Additionally, the initial stage of postsecondary life is a critical period in students’ development. In the transition from high school to postsecondary education, first-year students experience many changes in their internal and external environments (Hintz et al., 2014; Wei et al., 2014). These changes tend to place first-year postsecondary students at risk of Internet addiction. Therefore, we selected first-year postsecondary students as our subjects.

## Literature Review

### Social Support and Loneliness

Social support refers to the material and spiritual care, help, and support from others during difficult times or emergencies (Sherbourne and Stewart, 1991). Social support is generally believed to be divided into two dimensions according to its nature (Kessler et al., 1985). One is objective support (OS), which refers to objective, visible, or practical support. Such support is independent of individual feelings and is an objective fact (Thoits, 1983). For example, living with family, classmates, colleagues, or friends (Xiao, 1994). The other is subjective support (SS), which refers to the subjective and experiential emotional support of individuals (Thoits, 1983). For example, care and help from friends. Moreover, Xiao (1994) proposed that social support should include support utilization (SU); for example, taking the initiative when in trouble to inform friends to obtain their support and understanding. Thus, we proposed that when evaluating social support, the utilization of support should be a third dimension of social support.

Loneliness is a subjective psychological feeling or experience that occurs when an individual senses a lack of satisfactory interpersonal relationships and a gap between his or her desired and actual levels of communication (Ditommaso et al., 2003). Peplau and Perlman (1979) believed that loneliness is an unhappy experience related to an individual’s lack of social networks, including a lack of quantity and quality of social relations. Some researchers have demonstrated that social skills and coping methods for negative emotional events as well as social support, especially from core family members, rather than demographic variables such as sex, age, marriage, occupation, educational level, family, economic conditions, and socioeconomic status, substantively influence loneliness (Wu, 2004; Levitt et al., 2005). Numerous studies have demonstrated that in stressful situations, those who are psychologically or materially supported by a partner, friend, or family member are less likely to feel lonely (Chen et al., 2007; O’Donovan and Hughes, 2007; Yildirim and Kocabiyik, 2010; Wu et al., 2016). Although many studies have confirmed a negative correlation between social support and loneliness, few have explored the relationship between the three dimensions of social support and loneliness (Xiao, 1994; Wu et al., 2016). Some researchers reported that individual differences exist in the utilization of social support; although some people are able to receive social support, they may refuse it (Xiao, 1994). Thus, examining the relationships between the three dimensions of social support and loneliness is necessary to understand them.

### Loneliness and Internet Addiction

Generally, researchers believe that loneliness is a crucial factor when considering Internet addiction (Morahan-Martin and Schumacher, 2003; Manouchehr et al., 2007; Song et al., 2014; Demir and Kutlu, 2016). Three relevant theories exist: Internet addiction leads to loneliness, loneliness leads to Internet addiction, or loneliness and Internet addiction interact with each other. People who hold the first view argue that heavy Internet use isolates Internet users from the real world (Manouchehr et al., 2007). Such users develop a hypocritical and fragile network of relationships at the expense of real-world relationships; therefore, loneliness is a by-product of excessive Internet use (Underwood and Findlay, 2004). This view was supported by Kraut et al. (2002), who reported that excessive Internet use increases loneliness and depression and reduces the volume of social relationships and psychological well-being.

The second view suggests that lonely people are more likely to be attracted to Internet use (Morahan-Martin and Schumacher, 2003; Özdemir et al., 2014; Song et al., 2014; Demir and Kutlu, 2016; Sharifpoor et al., 2017). Excessive Internet use occurs because the Internet provides a wider social network and a variety of online forms of communication. Lonely people are attracted to interactive social activities facilitated by the Internet that provide a sense of belonging, friendship, and communication. This view is consistent with the model proposed by Shapira et al. (2000), who suggested that lonely people spend more time online (Shapira et al., 2000). The third view has been confirmed by scholars such as Koyuncu et al. (2014) and Tian et al. (2017). They have claimed that the associations between loneliness and Internet addiction are bidirectional: Internet addiction and loneliness positively predict each other across time.

However, a unanimous conclusion has not been reached on this topic. This may be partly caused by the influence of the established model and selected methods. When discussing the relationship between loneliness and Internet addiction, several dimensions of Internet addiction should be determined. Specifically, the factors to be included are as follows: First, compulsive Internet use (CIU) and withdrawal from Internet addiction (WIA), which relate to a state of involuntary Internet use and the associated symptoms from withdrawing or reducing Internet usage, such as depression and anxiety (Young, 1997; Chen et al., 2003). Second, tolerance of Internet addiction (TIA), which means when people require more Internet content or must extend their Internet use to obtain the equivalent level of satisfaction that they received previously (Young, 1997; Chen et al., 2003). Third, time management problems (TMPs), which are the negative effects of excessive Internet use on study and work (Goldberg, 1995; Chen et al., 2003). Last, interpersonal and health problems (IHPs), which are the negative effects of excessive Internet use on a person’s social life and health. Although numerous studies have shown that a positive relationship exists between loneliness and Internet addiction, few studies have explored the relationship between loneliness and the four dimensions of Internet addiction. Moreover, no clear agreement exists on whether Internet addiction leads to loneliness, whether loneliness leads to Internet addiction, or whether loneliness and Internet addiction interact. Therefore, this study investigated the association between loneliness and the four dimensions of Internet addiction using a longitudinal cross-lagged model.

### Social Support and Internet Addiction

Traditional friendships based on face-to-face communication are generally believed to provide social support, social identity, and a sense of belonging (Hamm and Faircloth, 2005). Some researchers have shown that when social resources are relatively scarce, some individuals choose the Internet as a medium through which to meet their needs and gain social support, especially when they are required to reestablish social networks and communication with others because of environmental changes (Aspden and Katz, 1998; Corinna and William, 2007; Kang et al., 2013; Yao and Zhong, 2014). Many researchers have reported that people often play different roles in online games, which enables teenagers to obtain social support unavailable in real life and meet various emotional needs (Young, 1997; Trepte et al., 2012; O’Connor et al., 2015). However, some researchers believe that relationships formed on such networks are shallow, illusory, and sometimes risky and hostile (Hills and Argyle, 2003; Wei, 2010; Fusco et al., 2015). In conclusion, whether the relationship between social support and Internet addiction is positive or negative as well as whether other variables (e.g., loneliness) exist between them is unclear. Dividing social support and Internet addiction into several dimensions as previously described helps to clarify the relationships between them. Therefore, determining the causation between the three dimensions of social support and the four dimensions of Internet addiction is necessary to understand the mechanism of Internet addiction.

### Limitations in Previous Researches

Although a growing body of work explicates the relationships among social support, loneliness, and Internet addiction (e.g., O’Donovan and Hughes, 2007; Yildirim and Kocabiyik, 2010; Song et al., 2014; Yao and Zhong, 2014; Demir and Kutlu, 2016), few studies have explored the relationships among loneliness, the three dimensions of social support, and the four dimensions of Internet addiction. Furthermore, several limitations exist in what has been accomplished, which has revealed the necessity for further studies.

First, research addressing the directionality of associations among pre-existing psychosocial variables (e.g., social support and loneliness) and Internet addiction is limited (Wu et al., 2016). A study examined the causation between Internet addiction and loneliness (Yao and Zhong, 2014); the results indicated that prolonged and unhealthy Internet use increases the severity of loneliness over time (Yao and Zhong, 2014). However, the time span between their two surveys was too short to detect a stable change in Internet addiction and loneliness over time or to examine the dynamic association between them. Excessive and unhealthy Internet use may increase feelings of loneliness, and increased loneliness may further increase excessive and unhealthy Internet use (Koyuncu et al., 2014; Tian et al., 2017). In addition, their study did not examine whether other antecedent variables (e.g., social support) affect loneliness. Second, Studies on social support, loneliness, and Internet addiction have mostly applied a cross-sectional design (e.g., Wu et al., 2016; Sharifpoor et al., 2017), which can reveal correlations between variables but cannot distinguish causal relationships.

### Overview of the Present Research

A cross-lagged structural equation modeling (SEM) design was used to investigate the associations among the three dimensions of social support and loneliness and the four dimensions of Internet addiction in first-year postsecondary students. A key objective of the present study was to establish causation using cross-lagged regression coefficients to compare these associations with respect to their strength, as well as to investigate the cause-and-effect relationship between social support, loneliness, and Internet addiction. The strongest association was determined to identify the most relevant causal influence driving the system. Additionally, the autoregressive effects of the model indicated the stability of the individual differences in scores over time.

Based on the aforementioned discussion and literature review, we put forward the following research purposes: (a) Explore the relationship between the three dimensions of social support and loneliness over time. (b) Explore the relationship between Loneliness and the four dimensions of Internet addiction over time. (c) Explore the causal relationships between loneliness, the three dimensions of social support, and the four dimensions of Internet addiction. (d) Answer the question of whether loneliness plays a mediating role in the relationship between the three dimensions of social support and the four dimensions of Internet addiction.

## Materials and Methods

### Participants and Procedure

The participants were recruited from a typical postsecondary school in Shandong Province, China, using cluster random sampling. The questionnaire measurements were taken at the beginning of the school year (T1), 6 months later (T2), and 1 year later (T3). This research was performed in accordance with the code of ethics of the World Medical Association (Declaration of Helsinki) for experiments involving humans and was approved by the Ethics Committee of Shandong Normal University. Besides, our research was obtained the written informed consent from participants. In the first measurement, a total of 220 adolescents were enrolled. Overall, 214 students responded to the questionnaire (the response rate was 97.27%), comprising 103 boys (48.13%), and 111 girls (51.87%). Of the initial sample, 13 students did not complete the second measurement and 19 students did not complete the third measurement because of sickness or other incidences. Additionally, 13 students provided invalid responses (e.g., the participants responded with the same pattern “1, 2, 3, 4, 4, 3, 2, 1, 1, 2, 3, 4...” or “1, 1, 1, 1, 1, 1...” in response to a 4-point scale). Consequently, the final sample comprised 169 students (88 girls and 81 boys), with an average age at the beginning of the study of 18.31 years (standard deviation [*SD*] = 0.77). A series of independent sample *t*-tests was conducted to determine whether participating adolescents differed from non-participating adolescents regarding any of the variables applied in the study. As shown in **Table 1**, none of the results from this analysis were significant.

**Table 1:** Independent sample *t*-tests on participating and non-participating adolescents.

Variables	P1-3 (*n* = 169)		P1 (*n* = 13)		*df*	*t*	P2 (*n* = 19)		*df*	*t*
	*M*	*SD*	*M*	*SD*			*M*	*SD*		
1. OS (T1)	6.06	1.75	6.62	1.39	180	-1.37	5.55	1.88	186	1.13
2. SS (T1)	19.48	2.45	18.21	4.36	180	1.69	19.81	2.63	186	-0.55
3. SU (T1)	8.47	1.77	9.23	1.83	180	-1.50	8.13	1.45	186	0.79
4. CIU & WIA (T1)	10.50	3.13	10.92	3.01	180	-0.47	10.26	3.36	186	0.31
5. TIA (T1)	7.79	2.35	8.00	2.89	180	-0.31	8.00	2.42	186	-0.37
6. TMP (T1)	9.30	3.25	9.69	2.84	180	-0.42	9.00	2.69	186	0.39
7. IHP (T1)	7.10	2.50	6.85	1.52	180	0.35	7.16	2.61	186	-0.10
8. Loneliness (T2)	39.48	9.03					38.79	8.21	186	0.32

*(a) OS, objective support; SS, subjective support; SU, support utilization; CIU & WIA, compulsive Internet use & withdrawal from Internet addiction; TIA, tolerance of Internet addiction; TMP, time management problems; and IHP, interpersonal and health problems. Similarly, hereinafter: (b) P1–3 represents the results of the participants who completed three-wave measurements; P1 represents the results of the participants who completed the first measurement only; and P2 represents the results of the participants who completed the first and second measurements*.

A team of researchers was established to collect data. Members of this team were trained and voluntarily involved. Data were collected through self-reporting. Students were informed that no one in their university would see the reports and the researchers would not know who the students were when processing collective reports. The students then signed informed consent forms voluntarily and the researchers selected the students who wanted to participate in the survey. We assigned a number to each student to matching the data collected at each time point while ensuring confidentiality. Social support and Internet addiction were measured at the beginning of the school year (T1) and 1 year later (T3), and loneliness was measured 6 months after the beginning of the school year (T2). The researchers told the students the purpose of the study at the end of the measurement process.

### Measures

#### Social Support

Social support was measured using the Social Support Rating Scale (SSRS) compiled by Xiao (1994). The SSRS contains 10 items with three dimensions: OS (3 items; e.g., “When you have encountered emergency situations, what sources of comfort and concern have you received?”), SS (4 items; e.g., “How is your relationship with your roommate?”), and SU (3 items; e.g., “How can you ask for help when you are in trouble?”). There are four corresponding option after each question (e.g., “How is your relationship with your roommate? 1 = We never care about each other, 2 = There’s only a little bit of care between us, 3 = Most of us are care about each other, 4 = Everyone cares about each other”). Participants choose the option according to their own situations. Relevant reports have demonstrated good reliability and validity for the scale (Liu et al., 2009; Tian et al., 2013). A high score in each subscale revealed high levels of OS, SS, and SU, respectively. The Cronbach’s α for the total SSRS scale in the present study at T1 was 0.67, and the values for the three subscales were 0.67 (OS), 0.62 (SS), and 0.61 (SU). The Cronbach’s α for the total SSRS scale in the present study at T3 was 0.74, and the values for the three subscales were 0.78 (OS), 0.69 (SS), and 0.61 (SU).

#### Loneliness

Loneliness was measured using the Chinese adaptation (Wang, 1995) of the University of California, Los Angeles Loneliness Scale (Version 3) (Russell, 1996). This scale contains 20 self-statements concerning the status of peoples’ interpersonal relationships. The questions are answered on a 4-point scale for how often (1 = *never* to 4 = *often*) participants feel the questions apply to themselves, with high scores indicating greater perceived loneliness; for example, “Do you often feel neglected?” The Cronbach’s α for loneliness at T2 was 0.91.

#### Internet Addiction

Internet addiction was assessed using an adaptation (Chen et al., 2003) of the Chinese Internet addiction scale (CIAS-R), a 19-item survey comprising four dimensions: CIA & WIA (e.g., “If I don’t surf the Internet for some time, then I feel depressed”), TIA (e.g., “Compared with the past, I must spend more time on the Internet to be satisfied”), TMP (e.g., “Because I’m addicted to the Internet, I have reduced the time in my routine for leisure activities”), and IHP (e.g., “I used to stay up all night surfing the Internet, resulting in lethargy during the day”). The responses were rated on a 4-point scale (1 = *strongly disagree* to 4 = *strongly agree*). A high score in each subscale revealed high levels of CIU & WIA, TIA, TMP, and IHP, respectively. The CIAS-R has been used widely in China and its internal consistency coefficients have been reported in other studies to be greater than 0.94 (Chen et al., 2009). The Cronbach’s α for the total CIAS-R scale in the present study at T1 was 0.90, and the values for the four subscales were 0.75 (CIU & WIA), 0.73 (TIA), 0.82 (TMP), and 0.66 (IHP). The Cronbach’s α for the total CIAS-R scale in the present study at T3 was 0.94, and the values for the four subscales were 0.83 (CIU & WIA), 0.79 (TIA), 0.85 (TMP), and 0.74 (IHP).

### Analysis Approach

#### Model Specification

Confirmatory factor analysis (CFA) tests of whether the relationship between a factor and the corresponding measure item conformed to the theoretical relation were designed by us. CFA was used as the test process of the measurement model. Subsequently, a structural model was used to measure the relationship between the variables. SEM was implemented using Mplus 7.0, and the indirect effects were assessed using the bootstrap method to test the aforementioned hypotheses. The maximum likelihood was applied to estimate parameters for the SEM.

Structural equation modeling integrates many advantages of traditional statistical methods and has become a widely used method of analysis. Checking the fitness of the model typically considers the *χ^2^* statistic. Because the *χ^2^* statistic is sensitive to sample size, some researchers have suggested that the model fits well when the ratio of *χ^2^* to *df* is less than 2 (Wen et al., 2004). Additionally, the model fit index was used as the primary criterion to evaluate the fitness of the model. Some studies have reported that the root mean square error of approximation (RMSEA), Tucker–Lewis Index (TLI), and comparative fit index (CFI) provide more efficient performance, whereas the maximum likelihood is used for parameter estimation (Hu and Bentler, 1999). When both the TLI and CFI are 0.9 or more, the model is acceptable (Bentler and Chou, 1987); when the RMSEA is less than 0.05, the model fits well; the model is basically acceptable when the RMSEA is between 0.05 and 0.10 (Steiger, 1990).

#### Evaluation of the Underlying Assumption of SEM

Basic descriptive statistical results were obtained from a preliminary analysis. Subsequently, we tested whether the data conformed to the SEM hypothesis. Generally, SEM assumes that each variable has complete data. Therefore, this study used the series mean to replace missing values. Because neither the skewness nor the kurtosis was significant for the variables, an assumption of a multivariate normal distribution of the observed data was satisfied.

## Results

### Descriptive Statistics and Difference Test

The means and standard deviations of loneliness, the three dimensions of social support, and the four dimensions of Internet addiction are presented in **Table 1**. Independent sample *t*-tests were conducted to test differences between sexes. The results showed that the SU reported by female students was significantly higher than that of the male students at T1 and T3. Furthermore, the TMP reported by male students was significantly higher than that of the female students at T1. Loneliness and all other dimensions of social support and Internet addiction at the same time exhibited no differences with respect to sex (**Table 2**). Additionally, the results of the paired sample *t*-tests showed that, in general as well as for the boys or girls, SS and the four dimensions of Internet addiction at T3 were significantly greater than those at T1 (**Table 3**). In general as well as for the girls, SU at T1 was significantly greater than at T3.

**Table 2:** Means and standard deviations of loneliness, the three dimensions of social support, the four dimensions of Internet addiction, and sex differences.

Variables	Total (*n* = 169)		Male (*n* = 81)		Female (*n* = 88)		*df*	*t*
	*M*	*SD*	*M*	*SD*	*M*	*SD*		
1. OS (T1)	6.06	1.75	5.90	1.77	6.21	1.72	167	-1.12
2. SS (T1)	19.48	2.45	19.24	2.72	19.71	2.15	167	-1.24
3. SU (T1)	8.47	1.77	7.95	1.68	8.94	1.73	167	-3.76***
4. CIU & WIA (T1)	10.50	3.13	10.94	3.38	10.10	2.83	167	1.76
5. TIA (T1)	7.79	2.35	8.01	2.40	7.59	2.29	167	1.78
6. TMP (T1)	9.30	3.25	9.99	3.18	8.67	3.21	167	2.67**
7. IHP (T1)	7.10	2.50	7.43	2.46	6.78	2.52	167	1.69
8. Loneliness (T2)	39.48	9.03	40.26	9.59	38.76	8.48	167	1.08
9. OS (T3)	6.35	2.11	6.20	2.27	6.50	1.96	167	-0.93
10. SS (T3)	21.80	3.06	21.78	3.60	21.81	2.48	167	-0.06
11. SU (T3)	8.12	1.91	7.67	1.95	8.54	1.79	167	-3.02**
12. CIU & WIA (T3)	12.18	3.69	12.44	4.02	11.94	3.36	167	0.89
13. TIA (T3)	8.73	2.56	8.73	2.71	8.73	2.43	167	0.003
14. TMP (T3)	10.94	3.43	11.11	3.42	10.78	3.44	167	0.61
15. IHP (T3)	8.21	2.65	8.40	2.69	8.05	2.62	167	0.86

*^∗∗∗^*p* < 0.001; ^∗∗^*p* < 0.01; ^∗^*p* < 0.05*.

**Table 3:** Comparisons of the three dimensions of social support and the four dimensions of Internet addiction using a three-wave test.

	Variables	T1 (*M, SD*)	T3 (*M*, *SD*)	*df*	*t*	Comparisons
Total (*n* = 169)	OS	6.06 (1.75)	6.35 (2.11)	168	1.84	
	SS	19.48 (2.45)	21.80 (3.06)	168	-10.90***	T3>T1
	SU	8.47 (1.77)	8.12 (1.91)	168	2.31*	T1>T3
	CIU & WIA	10.50 (3.13)	12.18 (3.69)	168	-6.44***	T3>T1
	TIA	7.79 (2.35)	8.73 (2.56)	168	-4.99***	T3>T1
	TMP	9.30 (3.25)	1.094 (3.43)	168	-6.58***	T3>T1
	IHP	7.10 (2.50)	8.21 (2.65)	168	-6.27***	T3>T1
Male (*n* = 81)	OS	5.90 (1.77)	6.20 (2.27)	80	-1.20	
	SS	19.24 (2.72)	21.78 (3.60)	80	-7.83***	T3>T1
	SU	7.95 (1.68)	7.67 (1.95)	80	1.17	
	CIU & WIA	10.94 (3.38)	12.44 (4.02)	80	-3.72***	T3>T1
	TIA	8.01 (2.40)	8.73 (2.71)	80	-2.43*	T3>T1
	TMP	9.99 (3.18)	11.11 (3.42)	80	-3.24**	T3>T1
	IHP	7.43 (2.46)	8.40 (2.69)	80	-3.21**	T3>T1
Female (*n* = 88)	OS	6.21 (1.72)	6.50 (1.96)	87	-1.41	
	SS	19.71 (2.15)	21.81 (2.48)	87	-7.59***	T3>T1
	SU	8.94 (1.73)	8.54 (1.79)	87	2.23*	T1>T3
	CIU & WIA	10.10 (2.83)	11.94 (3.36)	87	-5.43***	T3>T1
	TIA	7.59 (2.29)	8.73 (2.43)	87	-4.80***	T3>T1
	TMP	8.67 (3.21)	10.78 (3.44)	87	-6.03***	T3>T1
	IHP	6.78 (2.52)	8.05 (2.62)	87	-6.21	T3>T1

*(a) ^∗∗∗^*p* < 0.001; ^∗∗^*p* < 0.01; ^∗^*p* < 0.05*.

Subsequently, bivariate correlations were computed for all variable dimensions (see **Table 4** for the results). There are moderate correlations, ranging from 0.44 to 0.52, between each dimension of social support at T1 and that at T3. The associations between each dimension of Internet addiction at T1 and T3 indicated that cross-time correlations existed between them, ranging from 0.51 to 0.60. Moreover, the associations between loneliness, the three dimensions of social support, and the four dimensions of Internet addiction were as expected. Specifically, loneliness and the four dimensions of Internet addiction were positively, and the three dimensions of social support were negatively correlated with loneliness and Internet addiction. Finally, the associations between social support and Internet addiction were both concurrent and longitudinal.

**Table 4:** Correlations between loneliness, the three dimensions of social support, and the four dimensions of Internet addiction, according to a three-wave test.

Variable	1	2	3	4	5	6	7	8	9	10	11	12	13	14	15
1. T1 OS	1														
2. T1 SS	0.24**	1													
3. T1 SU	0.26**	0.38**	1												
4. T1 CIU & WIA	-0.06	-0.13	-0.07	1											
5. T1 TTLA	-0.01	-0.19*	-0.08	0.66**	1										
6. T1 TMP	-0.07	-0.19*	-0.15*	0.60**	0.54**	1									
7. T1 IHP	-0.09	-0.26**	-0.15	0.57**	0.53**	0.69**	1								
8. T2 loneliness	-0.15*	-0.24**	-0.31**	0.32**	0.25**	0.31**	0.30**	1							
9. T3 OS	0.44**	0.17*	0.23**	-0.10	-0.12	-0.11	-0.10	-0.21**	1						
10. T3 SS	0.12	0.52**	0.35**	-0.08	-0.16*	-0.21**	-0.25**	-0.30**	0.24**	1					
11. T3 SU	0.14	0.09	0.44**	-0.20**	-0.18*	-0.22**	-0.23**	-0.27**	0.27**	0.29**	1				
12.T3 CIU&WIA	0.02	-0.13	-0.05	0.51**	0.45**	0.47**	0.42**	0.27**	-0.09	-0.10	-0.27**	1			
13. T3 TTLA	0.07	-0.19*	-0.12	0.44**	0.51**	0.36**	0.39**	0.25**	-0.05	-0.18*	-0.29**	0.75**	1		
14. T3 TMP	0.03	-0.23**	-0.14	0.44**	0.46**	0.53**	0.44**	0.32**	-0.12	-0.22**	-0.32**	0.79**	0.72**	1	
15. T3 IHP	-0.04	-0.20*	-0.08	0.50**	0.46**	0.49**	0.60**	0.29**	-0.10	-0.15	-0.26**	0.70**	0.69**	0.71**	1

*(a) *N* = 169. (b)^∗∗^*p* < 0.01; ^∗^*p* < 0.05*.

### Cross-Lagged Associations Among Social Support, Loneliness, and Internet Addiction

**FIGURE 1:**
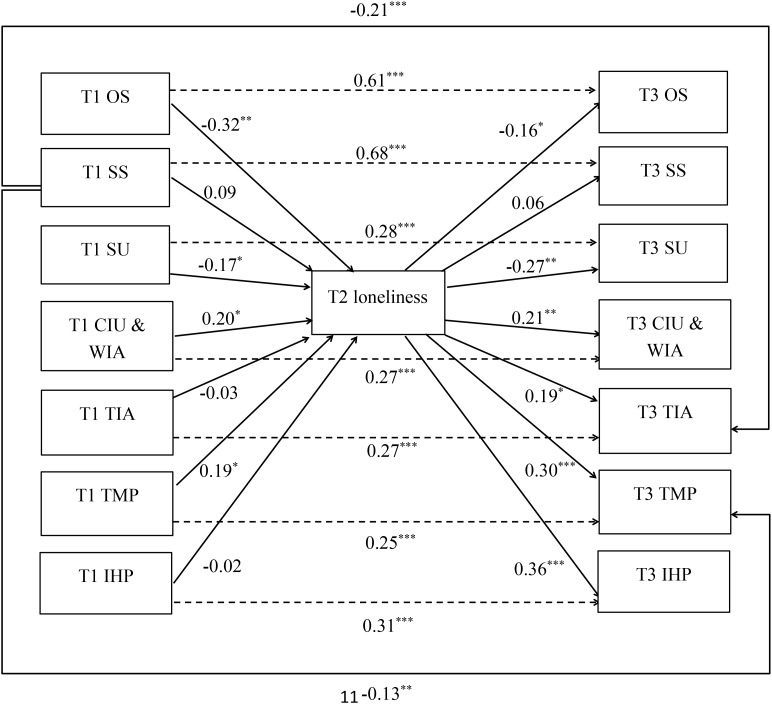
Cross-lagged associations among social support, loneliness, and Internet addiction. (a) *N* = 169; (b) ^∗∗∗^*p* < 0.001; ^∗∗^*p* < 0.01; ^∗^*p* < 0.05; (c) the solid line represents the regression between variables, and the dotted line represents the autoregression; (d) intercorrelations and paths between social support dimensions and Internet addiction dimensions at both time points are not shown in the figure for parsimony; and (e) the folding line in the figure represents the direct effect.

To assess longitudinal associations, a cross-lagged structural equation model was estimated with all three dimensions of social support predicting the four dimensions of future Internet addiction, as mediated by loneliness (see **Figure 1**). Because the results revealed significant differences by sex in the self-reported scores of SU at T3, we controlled the effect of sex on this variable in the following analysis. The model fitted the data well, *χ*^2^(47) = 90.73, *p* < 0.01, *χ*^2^/*df* = 1.93<2, CFI = 0.95, TLI = 0.90, and RMSEA = 0.08 (90% confidence interval [CI]: 0.05–0.10).

The results of the proportion of variance explained by the model are presented in **Table 5**.

**Table 5:** Results of the proportion of variance explained by the model.

	Loneliness (T2)	OS (T3)	SS (T3)	SU (T3)	CIU & WIA (T3)	TIA (T3)	TMP (T3)	IHP (T3)
R2	0.276	0.470	0.457	0.240	0.153	0.223	0.212	0.260

*N = 169*.

Structural equation modeling results revealed that each dimension of social support and Internet addiction at T1 predicted the same dimension at T3. Furthermore, OS and SU at T1 negatively predicted loneliness at T2. Loneliness at T2 positively predicted CIU & WIA, TIA, TMP, and IHP at T3. The indirect effect of OS (T1) to CIU & WIA (T3) through the mediating variable of loneliness (T2) was -0.07 (*p* < 0.05, 95% CI: -0.13 to -0.02), OS (T1) to TMP (T3) was -0.10 (*p* < 0.05, 95% CI: -0.17 to -0.02), OS (T1) to IHP (T3) was -0.12 (*p* < 0.05, 95% CI: -0.21 to -0.02), and SU (T1) to IHP (T3) was -0.06 (*p* < 0.05, 95% CI: -0.12 to -0.01).

CIU & WIA and TMP at T1 positively predicted loneliness at T2. Loneliness at T2 negatively predicted OS and SU at T3. However, the indirect effects revealed that loneliness at T2 was not a significant mediator of CIU & WIA (T1) to OS (T3) (99% CI: -0.11 to 0.004) or a significant mediator of CIU & WIA (T1) to SU (T3) (99% CI: -0.11 to 0.004). Additionally, no mediating effect occurred between TMP (T1) and OS (T3) (99% CI: -0.08 to 0.003) or SU (T3) (99% CI: -0.09 to 0.003). Finally, the direct effects of SS (T1) on TIA (T3) and TMP (T3) were significant. None of the other direct paths were substantial.

## Discussion

In this cross-lagged longitudinal study, the relationships between the three dimensions of social support and four dimensions of Internet addiction, as well as the mediating role of loneliness, were examined during the initial stage of postsecondary education. The results provided preliminary evidence of the relationship between the variables and suggested that loneliness plays a mediating role.

### The Relationship Among Loneliness, the Three Dimensions of Social Support and Four Dimensions of Internet Addiction Across Time

The two dimensions of social support (OS and SU) were negatively associated with loneliness and the relationships between them were bidirectional: OS and SU at T1 negatively predicted loneliness at T2, and increased loneliness negatively predicted T3 OS and SU. Numerous studies have demonstrated that a negative relationship exists between social support and loneliness (Wu, 2004; Chen et al., 2007; O’Donovan and Hughes, 2007; Yildirim and Kocabiyik, 2010; Wu et al., 2016). Therefore, the results of this study are consistent with relevant studies, and further confirm the interrelationship between social support and loneliness. Specifically, postsecondary students’ loneliness increased with a decline in social support (especially OS and SU). Lonely students may find it difficult to receive warmth and empathy from other people (Jobe and White, 2007; Pamukçu and Meydan, 2010), which may deter them from interpersonal interactions, reducing the likelihood of them being accepted by their peers, leading to lower social support.

Two dimensions of Internet addiction at T1, CIU & WIA and TMP, positively predicted loneliness at T2, and increased loneliness positively predicted all four dimensions of Internet addiction (CIU & WIA, TIA, TMP, and IHP) at T3. The results clarified that CIU & WIA and TMP are more likely to cause loneliness. Young and Rodgers (2009) indicated that extreme Internet use could cause loneliness; the results of our study confirm that assertion. People who experience withdrawal symptoms, such as depression and anxiety, after spending considerable time on the Internet may avoid socializing in real life, causing them to experience loneliness.

Conversely, loneliness positively predicted all four dimensions of Internet addiction. This indicated that loneliness has a stronger and more extensive effect on Internet addiction than Internet addiction has on loneliness. Anderson (2001) stated that people use the Internet to escape real problems and feelings of boredom, helplessness, anxiety, and depression. Loneliness is a sense of isolation and alienation from other people or from society (Chen et al., 2007; Wu et al., 2016), and is the self-closure of a person’s living space and a state of existence (Yildirim and Kocabiyik, 2010). Lonely people are divorced from social groups and live in a negative state (O’Donovan and Hughes, 2007), with this type of state making it easy for them to select networks to escape from real life (Anderson, 2001). In summary, loneliness and the several dimensions of Internet addiction positively predicted each other, and thus, produce a vicious circle.

### Unidirectional Causation Between Social Support and Internet Addiction

In the current study, we found that a unidirectional causation existed over time between the three dimensions of social support and four dimensions of Internet addiction. As the results demonstrated, the direct effects of SS (T1) on TIA (T3) and TMP (T3) were significant, and the indirect effects of OS (T1) on CIU & WIA (T3), TMP (T3), IHP (T3), and SU (T1) on IHP (T3) were significant. Specifically, SS, which refers to the subjective and experiential emotional support of individuals (Xiao, 1994), could directly predict the several dimensions of Internet addiction, whereas OS and SU affected the dimensions of Internet addiction through loneliness. This is reasonable, because no matter how much social support an individual receives through objective circumstances, the individual’s subjective and experiential feelings of social support pivotally affect his or her behavior (Stice et al., 2004; Moak and Agrawal, 2010). Students with low subjective social support are estranged from their family members, colleagues, and classmates (Gao et al., 2007; Sun et al., 2014); thus, they are prone to use the Internet to obtain comfort and satisfaction (Yao and Zhong, 2014).

In the relationship between OS/SU and the dimensions of Internet addiction, loneliness played a vital role. OS support and SU did not directly affect any dimension of Internet addiction, but they have indirect effects on Internet addiction through loneliness. Researchers have reported that problematic Internet use is a coping venue for limited social skills which result into regulatory difficulties (Caplan, 2002). Kardefelt-Winther (2014) proposed a conceptualization of internet addiction as a compensatory mechanism. Other researchers illustrated that problematic Internet-specific use relate to coping with low mood, and need to escape or gain status (Yao and Zhong, 2014; Lu and Yeo, 2015; Marino et al., 2016). Our study results are consistent with these studies, and further highlighted the mediating role of loneliness. Internet use has become one of the most common methods of solving loneliness and seeking psychological comfort (Song et al., 2010) for postsecondary students with low social support and feelings of helplessness and emptiness (Salehi and Seif, 2012). Furthermore, the virtuality and convenience of the Internet (Young, 1997), which does not exist in real life, make resisting the Internet difficult for postsecondary students with high levels of loneliness; thus, they frequently use it for satisfaction, which causes TMP as well as affects academic performance and other activities (Pang et al., 2010; Kim et al., 2017). Thus, the three dimensions of social support both directly and indirectly influenced the dimensions of Internet addiction through loneliness. Neither the direct or indirect effects from the four dimensions of Internet addiction on the three dimensions of social support were significant, which suggests that the causation between them over time is unidirectional.

### Other Notable Findings of This Study

In addition to the two main conclusions, there further notable findings were made. (a) Subjective social support and the four dimensions of Internet addiction at T3 were significantly greater than those at T1. (b) SU at T3 was significantly lower than that at T1. (c) Except for SU (T1 & T3) and TMP (T3), loneliness and all other dimensions of social support and Internet addiction at the same time exhibited no differences with respect to sex.

(a) and (b) indicate that subjective social support increased over time, whereas SU decreased. The reason for this may be that the students were unfamiliar with each other at the beginning of school, but with time, they became more familiar and intimate. Thus, when responding to items about subjective social support (e.g., “How many close friends do you have?” “How are relations between you and your roommates?”), their scores improved accordingly. The decrease of SU may be explained as follows. Participating in collective or school-organized activities is the main means for college students to utilize social support, and the items about social SU are also related to school-organized activities (Xiao, 1994). However, after 1 year of college, students lose their enthusiasm for these activities (Chen, 2005), leading to a decline in social SU.

Moreover, both subjective social support and Internet addiction levels increase over time. As students gradually adapt to college life, they have more leisure time. With convenient and constant Internet access through a variety of wireless devices, they often spend considerable time online and are therefore more likely to experience Internet addiction (Morahan-Martin and Schumacher, 2000). Studies have also shown that the Internet addiction level of college students increases with their year of study (Wang, 2006). Overall, the levels of subjective social support and Internet addiction of college students increase, but there is still a negative relationship between them. Therefore, this does not affect our research conclusions. The phenomenon of increasing subjective social support and Internet addiction suggests that there may be other variables than those considered in our study (i.e., social support and loneliness) that affect the Internet addiction of Chinese college students.

At the beginning of the college, as well as one year later, the SU of boys was significant lower than that of girls. This may be due to gender norms and specific concepts. Independence, autonomy, and self-sufficiency are typical male characteristics. On the contrary, typical female characteristics are interdependence, nurture, and caring for others (Cross and Markus, 1993). So girls are more likely to integrate into new groups and build new interpersonal relationships which help them to make good use of social support. Nevertheless, sex differences are not significant in the other two dimensions of social support. This is in line with previous research (e.g., Gao et al., 2007; Liu et al., 2009). Therefore, it can be said that our research has deepened the understanding of sex differences in social support. As for the sex difference in TMP at one year after beginning of the college, this may because boys are more willing to spend plenty of leisure time on games for entertainment than girls (Hupfer and Detlor, 2007; Niculović, 2014). However, on the whole, the sex differences of Internet addiction are not significant. So the Internet addiction of both college boys and girls deserves the attention of family, school and society.

## Implications for Preventing Internet Addiction

These findings have implications for the treatment and prevention of Internet addiction for first-year postsecondary students. First, the stronger the social support, the lower the likelihood a person will become addicted to the Internet. Therefore, as far as countermeasures are concerned, guidance toward the construction of first-year students’ social relations should be enhanced as much as possible. The degree of emotional experience that individuals have of being respected, supported, and understood in society is closely related to their subjective feelings, and thus, influences individuals’ attitudes toward social support and how they utilize it. Psychiatric studies have suggested that sufficient social support is beneficial to physical and mental health (Uchino, 2006). The Internet may be an alternative source of such support.

When students enter postsecondary education, their existing social connections may be altered or estranged, causing their social support to become unstable. Thus, they may require the reconstruction of new social networks. Researchers have demonstrated that Internet use is negatively related to social interaction and that increased Internet use may deepen the social alienation of users (Kraut et al., 1998). Therefore, either parents or the school can effectively help first-year students avoid developing Internet addiction by working to increase the social support of students, especially emotional support, and to enhance their interpersonal skills and the willingness to take advantage of social support. Helping boys integrate into the community as soon as possible is also very important considering their lower social SU.

Furthermore, Young and Case (2004) reported that education on and training against the risk of excessive or CIU can relieve many symptoms of Internet addiction. Internet addicts can list their activities that have been lost or neglected because of the Internet; these activities should be discharged according to the order of importance to improve the addicts’ awareness of real life. Additionally, the four dimensions of Internet addiction had a relatively high level of stability (0.27–0.44). Therefore, the treatment and prevention of Internet addiction should be performed as early as possible, at the beginning of postsecondary education.

## Limitations and Future Directions

Although this study made many contributions to understanding Internet addiction among first-year postsecondary students, several limitations should be addressed in future research. First, this study was conducted in a Chinese cultural context. Although this can help researchers understand and reveal cultural differences, exploring the universality of this problem is necessary in the future. For example, maladaptive cognition, motivation, and basic psychological requirements may cause differences in Internet addiction between adolescents in China and America (Hu et al., 2012). Some researchers have reported that the maladaptive cognition of Chinese adolescents includes both social facilitation and coping with stress, as well as the self-actualization dimension (Mai et al., 2012). Second, although self-reported assessments have several advantages for evaluating loneliness, social support, and Internet addiction, they also involve substantial limitations such as having demanding characteristics (e.g., confusing items, burden on memory, and requiring guesswork; Mitchell and Jolley, 1996). Besides, Cronbach’s alphas of social support are somewhat lower to the desirable ones, especially at T1. This reminds us that in data collection, we need a clearer and more accurate procedure to improve the reliability of measurement. Additionally, all variables were measured using self-reporting, which may have resulted in false or exaggerated correlations caused by common method bias. Future research should collect data from other sources of information, such as teachers, parents, or classmates, to test and verify the validity of the findings. Third, many studies have shown that loneliness has a multidimensional structure, such as peer and parent-related loneliness (Majorano et al., 2015; Corsano et al., 2017) and four-factor model of adolescent loneliness (Goossens et al., 2009). Therefore, future research can incorporate multidimensional loneliness into research and explore deeper relationships among variables. Fourth, despite the validity of the questionnaire measurements used in this study, additional psychological factors (e.g., self-control and impulsiveness) related to Internet addiction can be associated with the measurements and further examined. Fourth, we did not distinguish between various groups of Internet users. Some people are addicted to online games, whereas others are addicted to Internet pornography. Therefore, future research should test the proposed model among these various groups to clarify the mechanisms in varying forms of Internet addiction.

## Author Contributions

SZ, YT, and YS contributed to writing, data analysis, and finalizing the manuscript. DZ conducted the experiments and data analysis. JS, PW, WM, and YS also contributed to finalizing the manuscript.

## Conflict of Interest Statement

The authors declare that the research was conducted in the absence of any commercial or financial relationships that could be construed as a potential conflict of interest.
